# Telephone interventions in adherence to receiving the Pap test report: a randomized clinical trial ^1^

**DOI:** 10.1590/1518-8345.1845.2948

**Published:** 2017-12-04

**Authors:** Ana Izabel Oliveira Nicolau, Thaís Marques Lima, Camila Teixeira Moreira Vasconcelos, Francisco Herlânio Costa Carvalho, Priscila de Souza Aquino, Ana Karina Bezerra Pinheiro

**Affiliations:** 2 PhD, Assistant Professor, Via Corpvs, Centro Universitário Estácio do Ceará, Fortaleza, CE, Brasil; 3 PhD, Adjunct Professor, Departamento de Enfermagem, Universidade Federal do Ceará, Fortaleza, CE, Brasil; 4 PhD, Adjunct Professor, Departamento de Saúde Materno-Infantil, Universidade Federal do Ceará, Fortaleza, CE, Brasil; 5 PhD, Associate Professor, Departamento de Enfermagem, Universidade Federal do Ceará, Fortaleza, CE, Brasil

**Keywords:** Uterine Cervical Neoplasms, Telephone, Communications Media, Evidence-Based Nursing, Clinical Trial, Disease Prevention

## Abstract

**Objective:**

to test the efficacy of the behavioral and educational interventions undertaken by telephone, for women’s attendance at the consultation to receive the Pap test report.

**Method:**

a randomized clinical trial, with a sample randomized in three groups: telephone call - educational (n=171), telephone call - reminder (n=171) and comparison (n=169). The inclusion criteria were to be of legal age, to have become sexually active, to undertake the preventive examination during the study and to have a mobile or fixed telephone. The educational group received a telephone call involving a script based in the motivational interview and in the Brazilian guidelines. The behavioral group received a telephone call involving a reminder about the consultation. The comparison group received a card with details of when to return for a consultation regarding the results.

**Results:**

the women who received one of the interventions had a non-return rate of 7.3% and an increase of 39% (RR CI95%: 1.24-1.55) in the protection against this outcome. In the individual analysis of the interventions, it was evidenced that both are efficacious, as the telephone call - reminder reduces the woman’s failure to return to the service by 40% (RR CI95%: 1.25-1.57), while the telephone call - educational does so by 37% (RR CI95%: 1.22-1.54). The rates of non-return were of 6.5% and 8.2%, respectively, as against 33.1% in the comparison group.

**Conclusion:**

the interventions tested showed greater efficacy in the educational and behavioral contexts, in relation to the normal attendance, as they motivated the women to return to the service to receive the Pap test report. Clinical trial register: RBR-w3vnc.

## Introduction

In overcoming the challenges inherent in controlling cervical-uterine cancer, the nursing professional acts as a health promoting agent, a health educator, and a care provider. Her work covers all of the elements which are essential in the line of care for cervical cancer, which requires professional skills and abilities.

In the perspective of prevention, one finds an important obstacle referent to the difficulty of adherence and continuity of care: when the woman does not return to the health service, the following of the conducts related to the report does not take place in time, leading to a waste of time and resources on the part of both the service and of the woman, as the objectives of undertaking the examination - namely, prevention, early detection and empowerment, in relation to cancer, fail to be totally achieved. Furthermore, the opportunity is lost for a meeting between nurse and patient, which is an essential point for promoting guidance and clarifications in health ^1^. In primary health care, communicating the results of preventive examinations, along with the first follow-up measures, is undertaken mainly by the nursing professionals.

In an assessment undertaken in Brazil on failure to return for the consultation discussing the results (non-return), it was found that of 114 women, in the age range from 18 to 50 years old, and who were registered with the Family Health Strategy (ESF) in Ípora in the State of Goiás (GO), Brazil, 25% failed to attend^2^. A different situation was observed in Santa Maria in the Brazilian state of Rio Grande do Sul (RS), in which only 2% of the sample of 122 women - all from 25 to 64 years old - reported that they had not returned to the unit - this because a program reminding them to attend by telephone or post was already in place^3^. In the city of Fortaleza, in the Brazilian state of Ceará (CE), in a pilot-study undertaken in the Family Development Center (Cedefam, in Portuguese), based on the analysis of 3357 medical records from 2003 - 2011, a non-return rate of 23.2% was evidenced^4^, a situation similar to that of Iporá, GO.

Interventions have been described in the literature for increasing women’s adherence to the Pap test examination, in particular with a view to improving the information provided, to reducing barriers to accessing the examination, or both^5^ ^-^ ^6^. However: even in the light of these interventions’ efficacy, and of the high potential for curing cervical cancer, the prevention of this health issue can be compromised if the women fail to return to receive the examination’s results and, consequently, delay their adoption of the recommendations adjusted to each situation.

In the context of the interventions tested, in order to mitigate the issue of non-return, the main ones can be classified as behavioral, when they offer encouragement for undertaking the Papanicolaou examination and for returning to the unit to receive the results (reminders, for example) and as cognitive, which includes those aiming to provide further information and to educate the target-public ^(^ ^7^. Nurses’ recent efforts to increase the continuity of primary health care have included strategies such as issuing reminders by telephone (behavioral intervention) and telephone counseling (educational intervention), as a motivational possibility^8^ ^-^ ^9^.

The use of the telephone for motivating continuous care in health is often attractive, accessible and less onerous than other forms of action. Telephone interventions based in the literature and in the humanized approach, with space for specific questions about the examination, or even to discuss the patient’s fears, can be as efficacious for the continuity of the care as personally implemented interventions, and better than static means, such as letters, pamphlets and videos^10^ ^-^ ^11^.

The etiology of the problem related to non-return is multicausal, and may result from personal, social, educational or institutional reasons^1^. However, based on the premise that education can overcome these gaps and that - if undertaken in a participative and dialogic way - will culminate in empowerment, motivation and strengthening of women’s autonomy in conscious choices in health, it may be expected that women will adopt the stance of co-responsible subjects in adopting healthy behaviors. 

In the light of the above, the intention was to test the efficacy of the behavioral and educational interventions using the telephone for women’s attendance at the consultation where they receive the Pap test report.

The hypothesis to be tested is that “the application of educational or behavioral interventions by telephone, by the nurse, increases women’s adherence to attending the consultation provided when they return to receive the result of the Pap test examination”.

Studies of this nature are relevant, to the extent that they seek to value the following of the recommendations in time to achieve the real preventive power of the examination, to reduce unnecessary costs to the health system, and to encourage nurses to carry out efficacious strategies for controlling cervical cancer.

In the ambit of nursing’s scientific development, it is proposed, in this study, to present results with a high level of evidence in relation to telephone interventions, as yet unprecedented in the Brazilian context, regarding the problem of non-return. 

## Method

Study of the randomized clinical trial type, undertaken in the Lígia Barros Costa Natural Birth Center, run by Cedefam, situated on the outskirts of Fortaleza, CE, Brazil. Cedefam is linked to the Federal University of Ceará (UFC) for promoting health care for women and children by nurses and student nurses.

The study population was made up of women who had undertaken the cervical cancer preventive examination in the above-mentioned unit. The following inclusion criteria were used: to be 18 years old or over; to have initiated sexual activities; to undertake the Papanicolaou test in the data collection period, and to have a mobile or fixed telephone. The following were defined as exclusion criteria: to have some pathology related to mental processes, speaking or hearing, which would make it difficult to respond to the questionnaire and to participate in the interventions.

The reasons for losses to follow-up from the study were: not being possible to make contact via telephone in the groups in which there were interventions, or for the telephone conversation not to have been concluded. Telephone contact was considered to have failed only after three attempts, at different times of the day (during office hours), over two consecutive days. 

**Figure 1:**
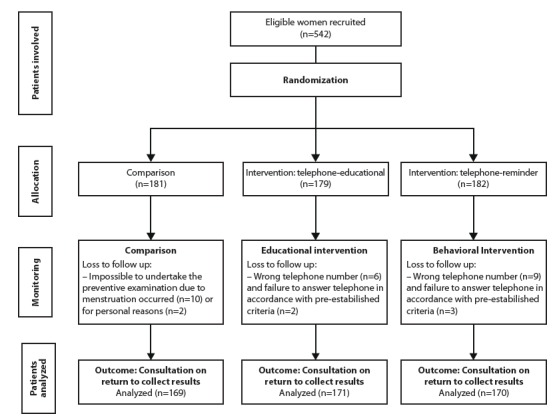
Flowchart of the conducting of the study. Fortaleza, CE, Brazil, 2014

For the sample calculation, the figure of 10% was used for clinically important difference. In the service selected, the non-return rate for collecting the report was 23.2%^4^. Upon inserting the values in the formula for studies with comparative groups (Zα=95%, Zβ=80%, p=23%, d=10%), the result was obtained that the study would need 139 women; however, for possible losses occasioned by the approach involving telephone contact (wrong number or change of number), a safety percentage of 30% was added, thus producing a total of 542 - approximately 180 women per group. At the end of the exclusions and losses to follow up, the sample was made up of 169 participants in the comparison group, 171 in the telephone call - educational intervention, and 170 in the telephone call - reminder intervention; thus totaling 510 participants, as shown in the flowchart in Figure 1. 

The method used for randomization had as its basis the women who received gynecological attendance - in order to determine the intervention groups (behavioral: telephone call - reminder/educational: telephone call-educational) and the comparison group. At the end of each month of data collection, the sample collected was compiled in an Excel spreadsheet, which contained the number of the medical record, each patient’s initials and age, preceded by the sequential number on the list. Once the data had been organized, random numbers were generated for the women’s random allocation to the three different groups in question. 

The participants were randomly allocated to three groups, namely: 

Group 1 - comparison: normal attendance (health education as found in waiting rooms, Pap test specimen collection and arranging the return visit through handing over a standardized sheet containing the date and time and name of the nurse responsible).

Group 2 - telephone call - educational intervention: as well as the normal attendance, the women were offered an intervention in the form of an educational telephone call, whose content was guided by the themes from script-file 4 from technology developed in a previous study^12^ and based in the Brazilian guidelines for controlling cervical cancer. The dialogue established in the telephone approach was conducted according to the principles of the Motivational Interview (MI). It is emphasized that MI has shown satisfactory results for changing behavior when used over the telephone and in chat groups on the Internet^13^.

The telephone call - educational intervention was divided in phases: the presentation of the researcher, building a relationship with the service user through open questions, requesting permission to provide information, initial soliciting of responses, exchanging of educational information and final soliciting of responses. In order to guide the conduct of the telephone call, a script was formulated in which the precepts of MI were carefully followed, in accordance with the stages mentioned above. These phases took place in a single telephone contact lasting approximately 10 to 15 minutes, one week prior to the date scheduled for the woman’s return to the unit. It is recommended that the intervention made over the telephone should last a maximum of 15 minutes^13^.

Group 3 - telephone call - reminder intervention: in addition to the normal attendance, a behavioral intervention was also offered (telephone call - reminder), whose content consisted of notice of the date and time of the consultation provided, one week before the date scheduled for the return to the unit. The behavioral intervention is based on the premise that people need encouragement (a reminder) to practice the appropriate conduct, as the behavior (response) is seen as the result of encouraged conditions^14^.

The telephone calls were made by two professional nurses (one for each intervention). These were blinded regarding the identity of the other professional and the other type of intervention tested, the two nurses having been trained separately. The professionals who were responsible for undertaking the tests were blinded as they did not know the disposition of the groups and of the interventions. In the initial consultation, the patients were not informed regarding the group to which they belonged - which also made possible the blinding of the patients. The evaluation of the main outcome (return to receive the results) was undertaken by the service’s team and by the researcher. There was no blinding in this, as this is a “hard” outcome, in which there is no uncertainty or possibility of biased evaluation, the outcome being, in this case, returning or not for the consultation. In the statistical analysis program used, the groups were named with letters (X, Y, Z) so as to make their identification by the statistician impossible and to make it possible for her to be blinded as well. 

The data were collected between June and December 2014. In the beginning of the monitoring process, the data collection instrument referent to the identification and to the questionnaire for evaluating Knowledge, Attitude and Practices (KAP), regarding both the examination and the importance of returning, was applied to all the women, while they waited for their consultation on prevention in one of the consulting rooms available in the health unit. The complete KAP questionnaire had been validated in a previous study^15^.

So as to ensure that the right to the consultation where the results are returned would be equal for all the women, all had their return to the unit arranged in a period of up to 40 days after the date when the examination was undertaken. In order to avoid the patients attended in the first months of data collection having a longer period of days for returning, a time period of up to 65 days was established (acceptable delay of up to a maximum of 25 days) for receiving the result. If the examinations were handed over after this period, they were, nevertheless, classified as “non-return”.

Monitoring was concluded in the return consultation, evaluating whether the woman returned on the day arranged and, if not, how many days there had been of delay. The interventions were evaluated through checking the women who attended to collect the report.

The data were compiled using the statistical program the Statistical Package for the Social Sciences (SPSS), version 20.0, and were later presented in tables and charts. The continuous variables were expressed as Mean (M)± Standard-Deviation (sd), with Confidence Interval (CI) of 95%. The categorical variables were expressed as frequencies and percentages. The groups were tested regarding homogeneity based on variance analysis tests (ANOVA) and the Pearson chi-squared test. In the assessment of the main outcome (percentage of women who returned) and of related factors, the Pearson chi-squared test and Relative Risk (RR) were used. The value of p≤0.05 was considered statistically significant. 

The clinical trial was described according to the international guide, Consolidated Standards of Reporting Trials (CONSORT) for nonpharmacological interventions^16^. The project was approved by the UFC’s Ethics Committee, under Opinion N. 700,619. The patients were invited to participate in the study, with later reading and explanation of the Terms of Free and Informed Consent (TFIC) by the researcher alongside the same, focussing above all on the objectives, procedures, risks and benefits of the three possible allocation groups. The clinical trial was registered under number RBR-3w3vnc in the Brazilian Clinical Trials Registry. The complete protocol for the clinical study is available electronically at: http://www.ensaiosclinicos.gov.br/rg/RBR-3w3vnc/.

## Results

A total of 542 KAP questionnaires were applied; however, it was not possible to make telephone contact with 20 patients, as can be seen in Figure 1. The main reasons were wrong number or nobody answering the telephone. In the comparison group, the losses occurred due to patients’ menstruation while they were waiting for the examination, or failure to return to collect the results for personal reasons (needing to return home due to some family problem, for example). 

The three groups were contrasted (Table 1) regarding homogeneity, so as to evaluate possible differences exceeding those expected as a result of chance and which might influence the outcome. 

**Table 1:** Characteristics relating to the start of the women’s follow-up, by group analyzed. Fortaleza, CE, Brazil, 2014

**Variable**		**Comparison group**		**Telephone call - educational group**		**Telephone call - reminder group**		**Test**
		M*±sd ^**†**^ **(CI** ^**‡**^ **95%)**		M*±sd ^**†**^ **(CI** ^**‡**^ **95%)**		M*±sd **(CI** ^**‡**^ **95%)**		**F** ^**§**^ **p** ^**||**^
**Age (years)**		**36.4±14.5 (34.2-38.6)**		**37.4±13.0 (35.4-9.4)**		**37.9±13.0 (35.9-39.8)**		**0.425**
**Educational level (in years)**		**9.2±3.1 (8.7-9.6)**		**8.9±3.5 (8.3-9.4)**		**9.3±3.2 (8.8-9.8)**		**0.544**
		**f**	**%**	**f**	**%**	**f**	**%**	**X** ^**2¶**^ **p**
**Marital status**								**0.573**
	**With partner**	**88**	**52.0**	**86**	**50.3**	**95**	**55.8**	
	**Without partner**	**81**	**48.0**	**85**	**49.7**	**75**	**44.2**	
**Has gynecological complaints**								**0.829**
	**Yes**	**78**	**34.5**	**75**	**33.2**	**73**	**32.3**	
	**No**	**91**	**32**	**96**	**33.8**	**97**	**34.2**	
**Cancer in the family**								**0.267**
	**Yes**	**68**	**40.2**	**78**	**45.6**	**63**	**37.1**	
	**No**	**101**	**59.8**	**93**	**54.4**	**107**	**62.9**	
**Participated in the educational group on cancer**								**0.101**
	**Yes**	**76**	**45.0**	**89**	**52.0**	**69**	**40.6**	
	**No**	**93**	**55.0**	**82**	**48.0**	**101**	**59.4**	
**Undertaking of the examination**								**0.248**
	**First time**	**8**	**4.7**	**3**	**1.8**	**8**	**4.7**	
	**Subsequent**	**161**	**95.3**	**168**	**98.2**	**162**	**95.3**	
**Returned for collecting results of last test**								**0.513**
	**Yes**	**139**	**86.3**	**138**	**82.1**	**139**	**85.8**	
	**No**	**22**	**13.7**	**30**	**17.9**	**23**	**14.2**	

*Mean; †Standard-Deviation; ‡Confidence Interval; §ANOVA; ||Level of significance; ¶Chi-squared test

In the evaluation of the main outcome (Table 2), of the total number of women who undertook the preventive examination, 429 (84.1%) returned to collect the result. 

**Table 2:** Distribution of the participants, according to whether they returned to collect the report on the Papanicolaou test. Fortaleza, CE, Brazil, 2014

**Variable**	**Comparison group** **(n=169)**		**Telephone call - educational group (n=171)**		**Telephone call - reminder group (n=170)**		**Total** **(N=510)**	
	**f**	**%**	**f**	**%**	**f**	**%**	**f**	**%**
**Returned**	**113**	**66.9**	**157**	**91.8**	**159**	**93.5**	**429**	**84.1**
**Did not return**	**56**	**33.1**	**14**	**8.2**	**11**	**6.5**	**81**	**15.9**

The rates of non-return were 6.5% in the telephone call - reminder group, and 8.2% in the telephone call - educational group, the equivalent of a fifth of that found in the comparison group, where the percentage was 33.1% in the first case, and one quarter, in the second. Statistical differences were evidenced with the rates of comparison for the consultation in the group studied, as shown in Table 3. 

**Table 3:** Association of the groups, according to the percentage of attendance for the consultation where the results of the test were to be returned. Fortaleza, CE, Brazil, 2014

**Percentage of Return (%)**			X²*	**p** ^**†**^	**RR** ^**‡**^ **(CI** ^**§**^ **95%)**
**Telephone call - reminder or educational** **92.7**	*vs* ^**||**^	**Comparison ** **66.9**	**60.072**	**0.000**	**1.39 (1.24-1.55)**
**Telephone call - reminder** **93.5**	*vs* ^**||**^	**Comparison** **66.9**	**38.001**	**0.000**	**1.40 (1.25-1.57)**
**Telephone call - educational** **91.8**	*vs* ^**||**^	**Comparison** **66.9**	**32.769**	**0.000**	**1.37** **(1.22-1.54)**
**Telephone call - reminder** **93.5**	*vs* ^**||**^	**Telephone call - educational** **91.8**	**0.370**	**0.543**	**1.01 (0.96-1.00)**

*Chi-squared test; †Level of significance; ‡Relative Risk*;* §Confidence Interval; ||*versus*

Among the target-public of the educational or behavioral interventions, 7.3% did not return. As a result, the interventions - in general - presented a rate of 39% (RR/CI 95%: 1.24-1.55) efficacy for protection against non-return when compared with that of the comparison group. 

In the individual analysis of the interventions, it was evidenced that both are efficacious for motivating women to return to collect the results, as the telephone call - reminder prevents the woman’s failure to appear at the service in 40% of cases (RR/CI 95%: 1.25-1.57), while the telephone call - educational, does so in 37% (RR/CI 95%: 1.22-1.54). In the comparison of the two intervention groups, there was no statistical difference in the *X*² test, which confirmed the efficacy of both strategies, although the non-return rate in the telephone call - reminder group was slightly lower (1.7%).

## Discussion

The behavioral and educational interventions tested demonstrated efficacy, such that, in the end, the non-return rates were of 6.5% and 8.2% respectively, as against 33.1% in the comparison group, a value superior to that found - 23.2% - in the same service^4^. In a separate intervention study, undertaken in Fortaleza, the educational group (a flip chart in the waiting room) presented a non-return rate of 18%, while the behavioral group (who were given a bracelet with the return date printed on it) had a rate of 34%, and the comparison group, one of 23%. The bracelet for reminding the service users of the date did not bring any benefits, but, rather, contributed to distancing the women from the service ^12^. As a result, the interventions tested in the present study were shown to be more simple and efficacious for motivating the women to attend to collect the report.

To cause positive impacts, it is important that the educational approaches should be innovative and interesting, for both the professionals and the clientele; they must be consistent with the socioeconomic, cultural and educational needs and characteristics of the target-public; they must be feasible in the health services’ routines and thus, possible to undertake continuously rather than occasionally; they must be low-cost and require little time; must be broadly accessible so that it may be possible to reach a large number of people and, furthermore, must take into account the weaknesses, barriers, motivations and strengths of each individual in adopting healthy habits, rather than simply providing information.

In the interventions tested, these aspects are considered, including in the use of the telephone as an educational means which is practical, accessible and less onerous for the patients, in relation to other forms of action. One can assert, therefore, that the strategies implemented contributed to the idea that telephone calls are a further tool for holistic care, constitute evolution of traditional care, and do not constitute a barrier to personal contact between patient and professional^10^ ^-^ ^11^.

Through the telephone call - educational intervention, based in the clinical technique of MI, it was possible to elicit the patients’ good motivations, so that they would carry out the behavioral changes in the interests of their health, with results which were even more positive because they emphasized that they had an active role in their own care, thus removing them from the state of ambivalence^13^. The telephone approach, due to being a strategy with interactive dialogic spaces, is also useful for identification and reduction in educational, psychological and practical barriers, being revealed to be a method which is efficacious for adherence to gynecological care.

The efficacy of telephone counseling was also evidenced in Germany, in the context of mammography tracking^17^. In the educational intervention tested, as it followed a collaborative motivational approach, which is evocative and respectful to the patient’s autonomy, promising results were demonstrated, with percentage differences which were greater than those presented in the above-mentioned study, with a difference of approximately 25 percentage points (8.2 *vs* 33.1%; p=0.000) between the rates of non-return of the telephone call - educational intervention group, and the comparison group.

In the context of the behavioral interventions in the ambit of cervical cancer, the reminder given by voice, interactively, during the telephone call was shown to be efficacious for increasing the undertaking of the examination. The telephone calls - reminder are more efficient than printed reminders, as they promote greater awareness and consequent improvement in spontaneous participation in the healthcare^18^. In attendance to collect the report, in the present study, the non-return rate reduced through the use of the reminder over the telephone, by approximately 27 percentage points (6.5 *vs* 33.1%; p=0.000), which was shown to be extremely efficacious for the outcome studied. 

The use of the telephone, whether for reminding or counseling, is efficient, above all because it presents great efficacy in the light of the variety of the population’s characteristics, contexts and needs, being shown to be an accessible strategy with a positive impact, including among women with low educational or economic levels. In addition to this, in the context of attending the consultation where the results are returned, the element of “forgetting” is an important contributing factor to failure to return, being resolved through the interactive, simple, fast and low-cost intervention.

It becomes relevant to undertake studies evaluating the perceptions and suggestions of the patients themselves regarding telephone-based strategies, or even to investigate managerial practices in search of methods which are favorable to the incorporation of telephone-based interventions in the services’ routines. The evidence could be strengthened through the undertaking of further clinical trials investigating possible significant differences between the interventions tested.

The principal limitations in this study lay in the exclusion of patients younger than 18 years old and the short time period for assessing return (65 days). It is suggested, therefore, that studies should be undertaken including the adolescent public, and which monitor the participants for a longer period in evaluating the effect of the interventions. 

## Conclusion

The telephone-based interventions were evidenced as efficacious in the educational and behavioral context, in relation to normal attendance, for improving women’s attendance at the consultation provided upon their return to receive the result of the Pap test examination.

Besides the positive effect on the principal outcome evaluated, one can add - as a favorable point - the fact that interventions are simple to apply, require little time or financial resources, and cover women from different socioeconomic and educational levels.

## References

[1] Vasconcelos CTM, Cunha DFF, Pinheiro AKB, Sawada NO (2014). Factors related to failure to attend the consultation to receive the
results of the Pap smear test. Rev. Latino-Am. Enfermagem.

[2] Oliveira WMA, Barbosa MA, Mendonça BOM, Silva AA, Santos LC, Nascimento LCD (2012). Adesão de mulheres de 18 a 50 anos ao exame colpocitológico na
estratégia saúde da família. Rev Enferm Ref.

[3] Rocha BD, Bisognin P, Cortes LF, Spall KB, Landerdahl MC, Vogt MSL (2012). Exame de Papanicolaou: conhecimento de usuárias de uma unidade básica
de saúde. Rev Enferm UFSM.

[4] Cunha DFF (2014). Fatores de risco para a descontinuidade na detecção precoce do câncer de colo
uterino [dissertação na Internet].

[5] Bennett AT, Patel DA, Carlos RC, Zochowski MK, Pennewell SM, Chi AM (2015). Human Papillomavirus Vaccine uptake after a tailored, online
educational intervention for female university students: a randomized controlled
trial. J Womens Health.

[6] Sly J, Jandorf L, Erwin DO (2015). Who’s missing? Predictors of attrition following participation in
culturally targeted educational breast and cervical cancer outreach programs for
latinas. J Health Commun.

[7] Yabroff KR, Zapka J, Klabunde CN, Yuan G, Buckman DW, Haggstrom D (2011). Systems strategies to support cancer screening in U.S. primary care
practice. Cancer Epidemiol Biomarkers Prev.

[8] Sabatino SA, Lawrence B, Elder R, Mercer SL, Wilson KM, DeVinney B (2012). Effectiveness of Interventions to Increase Screening for Breast,
Cervical, and Colorectal Cancers. Am J Prev Med.

[9] Ricard-Gauthier D, Wisniak A, Catarino R, Van Rossum AF, Meyer-Hamme U, Negulescu R (2015). Use of Smartphones as Adjuvant Tools for Cervical Cancer Screening in
Low-Resource Settings. J Low Genit Tract Dis.

[10] Vasconcelos HCA, Freitas RWJF, Marinho NBP, Lima FET, Araújo TL, Damasceno MMC (2013). Effectiveness of telephone interventions as a strategy for glycemic
control: an integrative literature review. Texto Contexto-Enferm.

[11] Bortolon CB, Machado CA, Ferigolo M, Barros HMT (2013). Abordagem motivacional para familiar de usuário de drogas por
telefone: um estudo de caso. Contextos Clínic.

[12] Vasconcelos CTM, Pinheiro AKB, Nicolau AIO, Lima TM, Barbosa DFF (2017). Comparison among the efficacy of interventions for the return rate to
receive the Pap Test report: randomized controlled clinical trial. Rev. Latino-Am. Enfermagem.

[13] Rollnick S, Butler CC, Kinnersley P, Gregory J, Mash B (2010). Motivational interviewing. BMJ.

[14] Skinner BF (2004). Psychology in the year 2000. J Exp Anal Behav.

[15] Vasconcelos CTM, Pinheiro AKB, Castel ARP, Costa LQ, Oliveira RG (2011). Knowledge, attitude and practice related to the pap smear test among
users of a primary health unit. Rev. Latino-Am. Enfermagem.

[16] Boutron I, Moher D, Altman DG, Schulz KF, Ravaud P (2008). Extending the CONSORT statement to randomized trials of
nonpharmacologic treatment: explanation and elaboration. Ann Intern Med.

[17] Hegenscheid K, Hoffmann W, Fochler S, Domin M, Weiss S, Hartmann B (2011). Telephone counseling and attendance in a national
mammography-screening program a randomized controlled trial. Am J Prev Med.

[18] Glick SB, Clarke AR, Blanchard A, Whitaker AK (2012). Cervical cancer screening, diagnosis and treatment interventions for
racial and ethnic minorities: a systematic review. J Gen Intern Med.

